# Successful Outcome of Anterior Cruciate Ligament (ACL) Reconstruction by Hamstring Tendon for Anterior Cruciate Ligament Deficit Knee at a University Hospital: A Descriptive Cross-sectional Study

**DOI:** 10.31729/jnma.7149

**Published:** 2021-12-31

**Authors:** Rohit Shrestha, Sushant Kumar Khadka, Sangharsha Thapa, Manasil Malla, Ashkal Basi, Prabha Bhandari, Laxman Aryal, Binay Kandel, Umesh Adhikari

**Affiliations:** 1 Department of Orthopedics, Dhulikhel Hospital, Dhulikhel, Nepal; 2 Kathmandu University School of Medical Sciences, Dhullikhel, Nepal; 3 Department of Physiotherapy, Dhulikhel Hospital, Dhulikhel, Nepal

**Keywords:** *anterior cruciate ligament*, *arthroscopy*, *knee*

## Abstract

**Introduction:**

Anterior cruciate ligament is a commonly injured and reconstructed ligament in the knee. Unlike in urban areas where sports activities and road accidents are common mechanisms of injuries, the semi-urban and rural population has a different mode of injuries, needs, and expectations. This study explores the prevalence of successful outcome of anterior cruciate ligament reconstruction in by hamstring tendon for anterior cruciate ligament deficit knee at a university hospital.

**Methods:**

A descriptive cross-sectional study was conducted at Dhulikhel Hospital, Kathmandu University Hospital from 2018 February to 2020 January among patients having anterior cruciate ligament injuries after ethical approval. Whole sampling technique was used. Functional outcome was assessed with Lysholm scale at the end of at least one year. Data was analysed using Statistical Package for Social Sciences version 11. Point estimate at 95% Confidence Interval was calculated, with frequency and percentage.

**Results:**

Out of 66 anterior cruciate ligament reconstruction, 35 (59%) cases had successful outcomes. Excellent outcomes were seen in 9 (15%) cases and 26 (44%) had good outcomes. The mean Lysholm score was 84.

**Conclusions:**

Anterior cruciate ligament injuries were seen in heterogeneous populations during their activities of daily living or recreational sports activities. Anatomic anterior cruciate ligamentreconstruction with hamstring grafts provided good functional outcomes, especially among the young population. Our findings are similar to current studies on anterior cruciate ligament-reconstruction.

## INTRODUCTION

The Anterior cruciate ligament (ACL) provides anteroposterior and rotational stability at the knee joint and hence it is crucial for various activities of daily living (ADL) like running, climbing stairs, and turning around the corners.^1–5^ ACL is one of the most commonly injured and reconstructed ligaments in the knee.^6,7^

The available literature on ACL injuries primarily focuses on young active individuals involved in sports activities. The populations in a semi-urban and rural setup, however, have different modes of injuries, needs, and expectations.^8^ Their ADLs include going uphill or downhill carrying a load on their back which requires knee strength and coordination. The evidence on the outcome of ACL reconstruction on this population is not well documented and yet to be explored.

Hence, this study explores the prevalence of successful outcome of anterior cruciate ligament reconstruction in by hamstring tendon for anterior cruciate ligament deficit knee at a university hospital.

## METHODS

With ethical approval from Institutional Review Committee KUSMS (approval no 006/19), a descriptive cross-sectional study was carried out at Dhulikhel Hospital, Kathmandu University Hospital from 2018 February until 2020 January among skeletally matured patients (age 16 to 60 years range, both males and females). They were the people presented with features suggestive of knee instability and supported by Magnetic Resonating Imaging findings of ACL deficiency and received arthroscopic single-bundle ACL reconstruction (ACL-R) with ipsilateral hamstring autograft. The exclusion criteria were cases with multi-ligamentous injuries, acute ACL repairs, ACL injuries associated with fractures, and ACL reconstructed with grafts other than hamstrings. Written informed consent was taken from all the participants. Whole sampling technique was used.

Sample size was calculated using the formula

n = Z^2^ × p × q / e^2^

  = (1.64)^2^ × 0.76 × (1-0.76) / (0.09)^2^

  = 60.56

Where,

n= minimum required sample size for infinite population
Z= 1.64 at 90% Confidence Interval (CI)
p= prevalence based on a similar study done in the past, 76%^9^
q= 1-p
e= margin of error, 9%

Standard trans portal anatomic single bundle ACL-R was done with graft anchored at the femoral side with fixed loop Endobutton (Smith and Nephew) and at the tibial side with bio-absorbable Interference screw (Polylevolactic acid - Smith and Nephew) of appropriate dimension. A negative pivot shift test under anesthesia after ACL-R was taken as an immediate indicator for adequate stability.

Postoperative protocols included standard care for wound management along with active rehabilitation that included isometric knee exercises which were started from post-operative day one, supervised a range of motion as tolerated, and was targeted to achieve full active extension and 90 degrees of knee flexion by the end of the first week. Patients were discharged after they were able to weight bear and ambulate with crutches or walkers as required and climb stairs under supervision. Hinged knee braces were prescribed only in cases where patients were not able to cope with the early postoperative rehabilitation protocol. Crutches were weaned off progressively from the fourth to eighth week depending on the patients' tolerance and gait. Physiotherapy was continued to strengthen the limb and improve power, proprioception, and range of motion.

The functional outcome was measured with the Lysholm scale, which is a 100 point scoring system consisting of eight sections, namely: limp, using walking aids, knee locking, knee giving way, pain, swelling, climbing stairs, and squatting. Single hop test (hop index) for distance, which represents the ability of a person to jump a certain distance with the operated limb as compared to his / her contralateral limb expressed in percentage, was used to assess strength and coordination. Data was analysed using Statistical Package for Social Sciences (SPSS) version 11. Point estimate at 95% Confidence Interval was calculated, with frequency and percentage.

## RESULTS

Out of 66 anterior cruciate ligament reconstruction, 35 (59%) cases had successful outcomes. Excellent outcomes were seen in 9 (15%) cases and 26 (44%) had good outcomes. The mean Lysholm score was 84.

The male to female ratio was 1.2:1. Both right and left knees were equally involved. Most of the patients were farmers and household workers followed by students. The majority of the patients were from Kavrepalanchowk district in the mid-hilly region of the country. Road traffic accidents and sports were the common mechanisms of injury among males, whereas, twisted knee injuries while performing activities of daily living (ADL) were common among the ladies (Table 1). The majority of the people receiving ACL-R were young and middle-aged with a mean age of 30.4 years and a range of 17 to 51years. Twenty to thirty years was the common age group among males suffering ACL injuries while 30 to 40 years was the common age group in females. Thirty-three (50%) patients had medial meniscus injury and 15 patients had lateral meniscus injury where seven patients were common in both the menisci injury. All meniscus injuries were managed either with a repair or partial meniscectomy as deemed appropriate.

**Table 1:** Demographic and Epidemiologic Parameters (n = 66).

Gender	n (%)
Male	36 (54.5)
Female	30 (45.5)
**Side**	
Right	34 (51.5)
Left	32 (48.5)
**Profession**	
Students	21 (31.8)
Farmers & Household Work	22 (33.3)
Office worker	13 (19.7)
Others (shops..)	9 (13.6)
**Address (Districts)**	
Mid Hilly Regions	42 (63)
Kavrepalanchowk	25 (38)
Sindhupalchowk	5 (8)
Others (Bhojpur, Ramechhap, Syanja, Kaski, Pyuthan, and Tanahu)	12 (18)
Terai & Madhesh	9 (14)
Kathmandu Valley	15(23)
**Injury Mechanism**	
Fall-related	6 (9)
Road traffic accidents	14 (21.2)
Sports activities	16 (24. 3)
Twisted injury while ADL (like downhill walking with load at back)	30 (45.5)

The majority 25 (38%) of the patients were operated on within three months of their ACL injury. On average, patients needed five days of inpatient care for wound management and physiotherapy training before they could be discharged. One of the patients needed anterolateral ligament (ALL) reconstruction as he had a positive pivot shift test immediately after ACL-R. This patient was not included in the outcome analysis (Table 2).

**Table 2:** Details regarding Operative parameters.

Injury - Surgery Interval	n (%)
within three months	25 (38)
three to six months	22 (33)
six months above	19 (29)
Median hospital stay	5 days
The median diameter of the final Hamstring graft	8.0mm
Mean length of the graft	84mm
Mean length of Femoral tunnel	36mm
**Length of graft intraarticular**	
At least 25mm	54 (81.8)
More than 25mm	12 (18.2)
**Graft Fold**	
Triplicated	4 (6)
Quadrupled	10 (15.3)
Pentapled	46 (69.7) [Median]
Hexapled	6 (9 )

There were 59 (89.4%) patients available for final follow-up evaluation with a mean of 21 months (3312 months) after the ACL-R. The follow-up visits were significantly affected by the lockdown amid the COVID 19 crisis at the terminal part of the study. However, among the available candidates, the mean Lysholm score was 84, and a majority 26 (44%) of the patients were in a good category. Most of the patients had developed an equivalent range of knee movement as compared to the contralateral healthy side along with a negative pivot shift test at the follow-up.

Three patients had grade I positive pivot shift test and were managed conservatively. The mean hop index in the current study was 78%, which shows that patients with ACL reconstructed knees can hop on average of about 78% distance as compared to the contralateral (normal) side. The timing of ACL-R whether done within three months or three to six months or after six months did not show any difference in the functional outcome (Table 3).

**Table 3:** Outcome Analysis at least one year of follow up (n = 59).

Distribution of Lysholm scores	n (%)	
Excellent (>90 score)	9 (15)	Success Rate
Good (90 to 84)	26 (44)	(Excellent & Good) 35 (59%)
Fair (83 to 65)	21 (36)	
Poor ( = <64)	3 (4.5)	
Range of motion of knees		
Same as that of the contralateral knees	52 (88)	
Extension lag	3 (5)	
Flexion reduced	4 (6.8)	
Stability (post-op pivot shift test)	3 (5)	All Grade 1
Single Hop test for Distance (HOP INDEX)-Mean For ACL reconstructed Side X100 % For the contralateral (Normal)	78%	
Lysholm Score		
**Injury to ACL-R Duration**	**n (%)**	**Mean ± SD**
within three months	21 (35.59)	81.4 ±9.5
three to six months	21 (35.59)	83.6 ±8.7
after six months	17 (28.81)	84 ±7.6

Subgroup analysis revealed that outcome was significantly associated with occupation and age group of study population while there was no association among the gender and geographic distribution (Table 4).

**Table 4:** Subgroup analysis according to Lysholm Scores.

	Excellent & Good n (%)	Fair & Poor n (%)
**Occupation**		
Students	17 (28.81)	3 (5.08)
Office worker	8 (13.55)	4 (6.77)
Others (Shop)	5 (8.47)	4 (6.77)
Farmer and Housework	5 (8.47)	13 (22.03)
**Age (years)**		
≤30	25 (42.37)	8 (13.55)
>30	10 (16.94)	16 (27.11)
**Gender**		
Male	19 (32.20)	14 (23.72)
Female	16 (27.11)	10 (16.94)
**Geographic Distribution**		
Hilly Districts	21 (35.59)	18 (13.50)
Urban and Flat area	14 (23.72)	6 (10.16)

Per operative complications were noted in two cases. In one, there was a lateral wall blow out and this case was managed with an interference screw. In the other case, there was posterior and lateral wall blow out and this was managed with a femoral post. In five cases, tibial posts were used in addition to tibial interference screws for better graft security. All the cases were included in the study as this study did not differentiate on the type of graft fixation. No major intraarticular infection was noted during the study period. However, two cases presented with serosanguinous discharge at the tibial graft fixation site approximately one year later that needed local debridement and removal of fragments of interference screws. Three cases had grade I pivot shift, three had extension lag and four had flexion deficit at follow up and they were satisfied with conservative treatment with physiotherapy as they were the low demand patients.

## DISCUSSION

The current study was carried out in a semi-urban to a rural setting in the mid-hilly region of the country, which correspondingly indicates the geographic terrain and the lifestyle of the people in their activities of daily living. The majority of the study population were involved in farming and almost half of the study population had their injury while performing household activities like walking up or downhill carrying a load at the back which is a typical rural lifestyle in the Nepali context. Sherchan B, et al. conducted a retrospective study in one of the tertiary care hospitals in Kathmandu which showed road traffic accidents as the most common mode of injury whereas Karn N, et al. found fall injury as the most common mode.^9,10^ These differences seen among Nepali articles might be attributed to the differences in the population composition and the study settings. Road traffic accidents, injuries related to falls, and sports are predominant in urban settings which represented their study population. The current study showed no differences in gender distribution whereas the majority of articles have shown ACL injuries and subsequently ACL-R were more common in males. This could be explained by the population demography of the mid-hilly rural population that included a relatively larger female population.^9-12^

The time interval from injury to ACL-R in the current study was found to be similar to other studies when the cohort was arbitrarily divided into three groups. In a retrospective study done by Razi, et al. in Tehran, they found that 37% of patients were operated on within three months.^13^ However, a British study by Church S, et al. had 56% of patients operated within three months.^14^ The reason why the timing of ACL-R is interesting to consider is that functional outcome, chances of complications, and most importantly chances of meniscal injuries are related to the timing of surgery. There were significant increases in associated medial meniscus injury with a delay of ACL-R beyond three months in studies as shown by Sajjadi MM, et al. Church S, et al. and Fithian DC, et al. in their studies.^14-17^ Grossly, the current study agreed with the findings obtained in other available studies that medial meniscus was most often associated with an ACL injury.^9,13-15^ The current study showed, however, similar functional outcomes among the groups who were operated on in different periods. This inference could be more robust if the study could have larger populations in each subcategory to compare the outcomes.

Several pieces of literature have extensively studied the intraoperative parameters. Few studies had explored the femoral tunnel length and its application in ACL-R.^16-19^ Miller LC, et al. had studied the intra-articular graft length in cadaveric knees who had received ACL-R before.^20^ Though these parameters depend on many other technical considerations, in a standard practice they remain similar for a given population and the knowledge of standard values may help in the planning of the surgery. Given the paucity of information in the local context, the present study adds on knowledge of the intraoperative parameters like femoral tunnel length, intra articular graft length, the graft fold, and diameters obtained by hamstring graft in the average Nepali population, and that is also supported by the previous study.^9^ However, in absence of extensive studies, it would be premature to generalize yet.

The average Lysholm score of the study cohort was good. Excellent and good constituted about 60% of the study population while good and fair were seen in 80%. Subgroup analysis revealed excellent and good category had more students and were of the younger population while people of age more than 30 years and occupation as farmer and household worker were more in the fair and poor category. This was just a crude observation and lacked enough evidence to derive a firm inference, however, differences in outcome may be attributed to multiple parameters like availability of rehabilitation supervision, early back to household activities, noncompliant with follow-ups, physical demand, and early or late weaning from the assistive device just to name a few of them. Interestingly the functional outcome was similar between the gender distribution and also the geographical location of the patient population. It could be argued that younger people and students have a better understanding of capacity, motivation, and access to proper post-op rehabilitation programs than their counterparts.^21^ Further research/studies would be appropriate to explore how the outcome results could be improved. The role of good physiotherapy and post-op rehabilitation cannot be underestimated.

The current study recognized certain limitations. The first was the use of the Lysholm score to measure the patient's reported functional outcome, which is yet to be translated and validated in local language and context for better credibility. The other was the need for larger samples and multi-center trials for firm inferences on cause and effect relationships. And finally, the current study did not segregate the associated meniscal injuries depending upon whether ACLR was done early or late, which would be a prospect for further study in the future.

## CONCLUSIONS

ACL injuries were seen in heterogeneous populations during their activities of daily living or recreational sports activities. Anatomic ACL-R with hamstring grafts provided good functional outcomes, especially among the young and active population. Our findings are similar to current studies. The geographic location of residence of the study population and the timing of ACL-R did not affect their functional outcome.
